# Editorial: The role of the interactions *via* movements in the spatial and temporal representation of external objects

**DOI:** 10.3389/fncom.2022.989724

**Published:** 2022-07-27

**Authors:** Daya Shankar Gupta

**Affiliations:** College of Science and Humanities, College of Health and Pharmacy, Husson University, Bangor, ME, United States

**Keywords:** perception, entropy, voluntary movement control, mutual information, muscle synergies, temporal, cognition

Humans execute movements to manipulate their physical surroundings to improve their survival chances. Successful interaction of the brain with the surroundings, which produces purposeful movements, depends on many factors, some of which are highlighted by the contributions in this special issue. The control of movements during an interaction with the physical environment is simplified by a grouping of muscles, called muscle synergies, which serve as the functional unit and can be used across task conditions (d'Avella et al., 2003). Muscle synergies are a small number of fixed patterns of contractions. Movements also reduce the entropy (a measure of surprise) in the patterns of the neuronal activities in the brain that will increase knowledge, forming the basis of voluntary control and perception (Gupta and Bahmer, 2019). In desynchronized states of the brain, which promote information processing (Petersen, 2019), the temporal coupling of neuronal events will occur as a result of the interactions with the physical world (Gupta et al., 2020; Gupta and Bahmer, 2021). Transferring the temporal relationship between external physical events, namely sensory stimuli and movements during an interaction, separated by zero to hundreds of milliseconds, to a corresponding temporal relationship between the neural events triggered by those external physical events requires an accurate representation of the time axis in the brain. This transfer of temporal relationships will lead to the temporal coupling of neural activities, given the external events involved in an interaction. Thus, a “successful interaction” will reduce entropy in activity patterns when neuronal activities are temporally coupled. This reduction in entropy or surprise will result in the gain of knowledge about the interaction, responsible for the sensorimotor control by the brain. Thus, humans control movements as they are being executed, which is referred to as online control (Oostwoud Wijdenes and Medendorp), by resolving the surprise in the spiking pattern of the cortical areas *via* temporal coupling of neuronal activities, given the external events involved in the interaction.

Online control depends on the instantaneous estimate of the current state of the arm and body in the world. According to Optimal Feedback Control theory, this estimate, which is modulated by context and shaped by experience, is based on integrating forward motor predictions and sensory feedback, such as proprioceptive, visual, and vestibular information. To simplify difficulties, inherent in understanding multimodal estimate's role in online control, Oostwoud Wijdenes and Medendorp have proposed that the earliest online movement corrections are based on multiple single modality state estimates rather than one combined multimodal estimate. Indeed, it has been argued in the past that the detection of visual stimuli pertaining to movements is a fundamental process for the control of reaching movements (Reichenbach et al., 2014).

The visuomotor response in a task involving interaction with physical surroundings is likely to involve both hemispheres, which is underscored in a study by Hagio and Kouzaki, which showed that a visuomotor perturbation during a barrier crossing task affects the movement of the trail leg in addition to the movement of the lead leg.

In their paper, Kostyukov and Tomiak simulate the shoulder and elbow joint torques (JTs), using a two-segment model of the human arm. In contrast to dynamic models, in which the second-order differential equations define the velocities and accelerations of different limb segments, the authors use steady states of the motor system in forced interactions as the chief elements of analysis. It is assumed that the CNS defines the equilibrium states in the motor interaction of the organism with the environment, while movements result from the transitions between a series of equilibrium states. Authors hope that a pattern of the torque effects can provide some simplification of both descending motor programs and their integration at the spinal level.

Josa et al. have studied the effect of action constraints on distance estimation. Authors report that subjects' distance estimation from a cart to a target depended on the weight of the cart, loaded with books or empty. The subjects overestimated the distance when the cart was loaded with books. This, the authors argue, is consistent with the embodied perception theories, which suggest that perception depends on the constraints of potential action.

Avraham et al. (B) investigated the effect of applying a 150 ms delay in visual feedback from the left visual workspace on lateral movements and visual perception of the mid-point of horizontal lines. The authors observed hypermetric movements on the left side, which returned to the baseline during adaptation. In another study by Avraham et al. (A), also with visual feedback of hand movement (movement of the cursor) was delayed by 150 ms, in right or left or both workspaces. The hand movement was followed by drawing circles in the desired direction without any visual feedback. Avraham et al. observed that “delay presented in left and both delay caused symmetrical elongation only to left initiated circles and right delay caused symmetrical elongation to both left and right initiated circles.” Both works underscore the importance of the representation of the time-axis in voluntary movements of the right hand. Presenting the delay in visual feedback also shifts the representation of time-axis, which could be responsible for hypermetric or elongated movements. These findings also suggest that a representation of the time-axis in the left hemisphere may be responsible for the laterality of hemispheres since the delay of the visual feedback in the right workspace (processed by the dominant left hemisphere) leads to symmetrical elongation of the left and right initiated circles.

Sorrentino et al. studied the development of spatial memory in children aged 4–6 years, which required collecting nine colored balls in buckets arranged in three different configurations, namely, Matrix, Cluster, and Cross. The trial ended when all nine colored balls were collected or 30 visits, wrong or correct visits were made, including revisits. The authors found that declarative spatial memory improved with age and movements. Findings showed spatial ability depended on the complexity of the environment.

Saccades are rapid movements of the eyes that abruptly change the point of fixation (Purves and Williams, 2001) when scanning the space during navigation. Fixation points during scanning of the scenery image are the most salient locations. Chauhan et al. present two versions of hierarchical Saccade Velocity Driven Oscillatory Network models. In these two hierarchical models, the output of one layer is used as input for computing the output of the next layer. First, a saccade trajectory map is generated according to decreasing order of saliency of different locations of an image. The saliency is based on three features: intensity, color, and orientation. The saliency trajectory map is processed by the saccade direction layer, which computes the animal's current saccade direction projection on the preferred direction. The computation in the next layer, called the path integration layer, incorporates an amplitude function, resulting in oscillations. The path integration layer projects to the output SC layer, which exhibits a grid-like pattern by extracting the principal components of the oscillatory response. The periodicity in the weights of the principal component due to oscillations corresponds to grid cells in the entorhinal cortex that fire action potentials in navigating animals. The authors argue that oscillations are critical for grid cell generation. Oscillations encode the position information in their respective phase. This is also supported by animal studies that showed a key role for theta oscillations in the normal grid cell activity in the entorhinal cortex (Giocomo et al., 2007).

Gundavarapu et al. present a hierarchical neural field network model of motion processing. The model architecture has an input layer followed by either one or two neural fields (NF), NF1, and NF2, corresponding respectively to the primary visual cortex and middle temporal area. In this model, the lateral connections in the neural fields are trained by unsupervised asymmetric Hebbian learning, to process sequential information in motion stimuli. Neurons in NF1 respond to the direction of the component motion, such as gratings and edges, and the neurons in NF2 respond to the direction of motion of the whole pattern. Additionally, information about the stimulus's temporal sequence is preserved in the network dynamics. Interestingly, translational random dot stimuli flow motion was decoded by a classifier with an accuracy of 90% on the test data, which is a biologically plausible range for most human activities involving interactions with moving objects. The success of neural networks in the above neurobiologically plausible models (Chauhan et al.; Gundavarapu et al.) suggests that many functions of the brain are due to hierarchical processing of information by different higher brain areas and may be driven by learning from experience, similar to neural networks.

Krüger and Hermsdörfer studied duration and fingertip position variability in reaching movements involving touching a target with the right index fingertip at a fixed distance. The targets were manipulated according to three conditions: forced choice with certain and uncertain targets, and a third free choice target. Consistent with previous literature, authors reported that “within-subject between trial variability of fingertip position showed an increase-decrease pattern across the time course of movement execution, with low variability at movement end.” The initial increase in the “between trial variability” is consistent with an increase in the entropy of neuronal spiking in cortical motor areas. However, near the end of the movement, coinciding with the interaction with the external world, there is reduced “between trial variability,” suggesting an increase in correlated activities, which is likely due to an increased probability of the task-specific activation of temporally coupled pairs of neurons. This time course of the change in variability from the beginning to the end of the task is consistent with the role of an initial increase in entropy followed by an increase in mutual information (a measure of correlation), representing information underlying purposeful action (Gupta and Bahmer, 2019). Furthermore, it is expected that the initial increase in the variability is greater if the number of targets is more than one. Thus, the authors found greater variability in fingertip position when two targets instead of one target were presented in a forced-choice task. Krüger and Hermsdörfer also reported an increased length of the movement path in the forced-choice task with two targets. We note that increased length of movement path in forced-choice task with two targets is likely related to greater variability and vice versa. Authors have argued that the increased length of the movement path is responsible for the significantly increased duration of the movement.

Min et al. used a computational model to argue that a learned motor skill can be adapted to a novel condition. The authors proposed that the use of a learned motor skill in a novel setting will produce feedback gain signals, which can tune the output of corticospinal neurons in a computational model of the cortico-basal ganglia-thalamic-cortical circuit. The basal ganglia dynamically modulate motor output with the synergistic combination of two control policies: group control policy (CGP) and individual control policy (ICP). The CGP represents all muscles controlled by a single peripheral nerve, and the IGP represents individual muscles. The synergy between two control policies is optimized by feedback gain signals according to the feedback signals to produce the movements adapted to a novel context. Feedback signals *via* the cortico-basal ganglia-thalamic-cortical circuit may help in monitoring the movement in a novel context for successful interaction. Learned motor skills may be stored in the premotor area as a circuit pattern, which may be activated in a novel context. The success of movements in novel conditions, given the use of learned motor skills, will be determined by the maximum decrease in entropy in overall cortical spiking firing patterns, which may depend on the temporal coupling of a slightly different set of pairs of neuronal activities. This may occur due to temporal coupling of neuronal activities, caused by signals from proprioceptors relayed by the cerebellum to the cortex as well as activities in cortical motor areas, reflecting actual interactions in novel conditions, which will lead to the modulation of the learned motor skill.

Cohn et al. have proposed the feasibility theory wherein a high-dimensional feasible activation space is a family of valid solutions representing muscle activation patterns, such as muscle synergies, for a given motor task. The authors argue that due to the dependence on anatomical constraints of the nervous system and musculoskeletal system, the feasible activation space contains valid solutions, i.e., prescriptive synergies for executing movements. Feasible activation space may also provide a framework for analyzing how learned motor skills may be modified in novel situations (Min et al.).

Oshima et al. present experimental evidence showing that humans adapt to different speeds of locomotion–walking and running–by altering the spatial coordination patterns, while the temporal coordination pattern remains unaffected by different speeds. Their findings based on the study of the motion of the legs indicate that the control of temporal patterns is independent of the control of spatial patterns. This is consistent with the identification of orthogonal components, which include patterns of muscle contractions, called synergies and temporal components from the analysis of electromyograms by standard multidimensional factorization algorithms (d'Avella et al., 2003). There may be different sets of patterns of muscle contractions–muscle synergies–at different speeds of locomotion, leading to altered spatial patterns. In a related study, Gonzalez-Rubio et al. observed that motor adaptation in the spatial domain was susceptible to feedback in the temporal domain, whereas motor adaptation in the temporal domain was not altered by the feedback in the spatial domain. Since invariant muscle synergies represent the spatial domain, there will be no effect of the feedback in the spatial domain. However, as suggested by other papers in this special issue, the neural representation of time-dimension can be dynamically updated during a task (Avraham et al., A; Avraham et al., B); thus, motor adaptation in the spatial domain is affected by feedback in the temporal domain.

Various contributions to this Frontiers Research Topic suggest that voluntary motor control depends on two independent components: (1) prior, such as muscle synergies, detected by electromyogram analysis, and (2) instantaneous sensorimotor interaction, which leads to temporal coupling of neural events in desynchronized brain states. Moreover, the temporal coupling of neural events depends on an accurate representation of physical time-dimension in the brain, in addition to sensorimotor interactions between the brain and physical surroundings. A successful sensorimotor interaction will result in the temporal coupling of neuronal activities that would reduce the entropy in the patterns of neuronal activities, given a particular task, contributing to smooth voluntary motor control. Future works should investigate how the temporal coupling of neuronal activities and different measures of surprise play a role alongside prior in voluntary movements.

## Author contributions

The author confirms being the sole contributor of this work and has approved it for publication.

## Conflict of interest

The author declares that the research was conducted in the absence of any commercial or financial relationships that could be construed as a potential conflict of interest.

## Publisher's note

All claims expressed in this article are solely those of the authors and do not necessarily represent those of their affiliated organizations, or those of the publisher, the editors and the reviewers. Any product that may be evaluated in this article, or claim that may be made by its manufacturer, is not guaranteed or endorsed by the publisher.
